# Combining Vγ9Vδ2 T Cells with a Lipophilic Bisphosphonate Efficiently Kills Activated Hepatic Stellate Cells

**DOI:** 10.3389/fimmu.2017.01381

**Published:** 2017-10-24

**Authors:** Xiaoying Zhou, Yanzheng Gu, Hongying Xiao, Ning Kang, Yonghua Xie, Guangbo Zhang, Yan Shi, Xiaoyu Hu, Eric Oldfield, Xueguang Zhang, Yonghui Zhang

**Affiliations:** ^1^School of Pharmaceutical Sciences, MOE Key Laboratory of Bioorganic Phosphorus Chemistry & Chemical Biology, Tsinghua University, Beijing, China; ^2^Jiangsu Key Laboratory of Clinical Immunology, Soochow University, Suzhou, China; ^3^Jiangsu Key Laboratory of Gastrointestinal Tumor Immunology, The First Affiliated Hospital of Soochow University, Jiangsu, China; ^4^Collaborative Innovation Center of Biotherapy, Sichuan University, Chengdu, China; ^5^Institute for Immunology and School of Medicine, Tsinghua University, Beijing, China; ^6^Department of Chemistry, University of Illinois at Urbana-Champaign, Urbana, IL, United States

**Keywords:** activated human hepatic stellate cells, liver fibrosis, Vγ9Vδ2 T cells, lipophilic bisphosphonates, hepatocellular carcinoma

## Abstract

Activated hepatic stellate cells (aHSCs) are now established as a central driver of fibrosis in human liver injury. In the presence of chronic or repeated injury, fibrosis, cirrhosis, and hepatocellular carcinoma (HCC) can occur, so there is interest in down-regulating aHSCs activity in order to treat these diseases. Here, we report that Vγ9Vδ2 T cells are reduced in patients with liver cirrhosis, stimulating us to investigate possible interactions between Vγ9Vδ2 T cells and aHSCs. We find that Vγ9Vδ2 T cells kill aHSCs and killing is enhanced when aHSCs are pretreated with BPH-1236, a lipophilic analog of the bone resorption drug zoledronate. Cytotoxicity is mediated by direct cell-to-cell contact as shown by Transwell experiments and atomic force microscopy, with BPH-1236 increasing the adhesion between aHSCs and Vγ9Vδ2 T cells. Mechanistically, BPH-1236 functions by inhibiting farnesyl diphosphate synthase, leading to accumulation of the phosphoantigen isopentenyl diphosphate and recognition by Vγ9Vδ2 T cells. The cytolytic process is largely dependent on the perforin/granzyme B pathway. In a Rag2^−/−^γc^−/−^ immune-deficient mouse model, we find that Vγ9Vδ2 T cells home-in to the liver, and when accompanied by BPH-1236, kill not only orthotopic aHSCs but also orthotopic HCC tumors. Collectively, our results provide the first proof-of-concept of a novel immunotherapeutic strategy for the treatment of fibrosis–cirrhosis–HCC diseases using adoptively transferred Vγ9Vδ2 T cells, combined with a lipophilic bisphosphonate.

## Introduction

Cirrhosis is an advanced stage of liver fibrosis and remains one of the central challenges in clinical hepatology. Fibrosis results from the excessive accumulation of extracellular matrix (ECM) proteins (e.g., collagen) at sites of tissue repair (1, 2). ECM proteins are produced by activated hepatic stellate cells (aHSCs), which represent ~10% of all liver cells (3). HSCs are activated following liver injury, and then transdifferentiate from quiescent lipocytes into ECM-producing myofibroblasts (4). This transdifferentiation process can drive fibrogenesis (4), and sustained fibrogenesis leads to cirrhosis. Thus, targeting aHSCs represents a potential approach for the treatment of heptatic fibrosis (5) and consequently cirrhosis. It has been shown that extracellular signals from immune cells, including macrophages (6), natural killer cells (7), natural killer T cells (8), and B cells (9) can modulate the activation of HSCs, but there are no reports in the literature that have addressed potential interactions between aHSCs and γδ T cells.

T cells that express the Vγ9Vδ2 T cell receptor comprise a small subset of the total T lymphocyte population. These so-called Vγ9Vδ2 T cells are centrally important in both innate and adaptive immune surveillance (10) where they act as first responders to confront many bacterial and protozoal pathogens. Activated Vγ9Vδ2 T cells can also kill cells in many types of tumors, and their use in “adoptive” immunotherapies is being investigated by several groups (11, 12). Vγ9Vδ2 T cells are unique to humans and primates and account for ~90% of the circulating γδ T cells in human blood. Importantly, it has been reported that there are decreased numbers of Vγ9Vδ2 T cells in patients with chronic hepatitis B (13) and hepatitis C (14), both of which are major risk factors for the development of liver fibrosis and hepatocellular carcinoma (HCC).

In a collaborative clinical research, we observed that Vγ9Vδ2 T cells were significantly reduced in cirrhosis patients, and this led us to hypothesize that Vγ9Vδ2 T cells function as immunosurveillance during the progression of cirrhosis by monitoring aHSCs. We thus sought to investigate: (1) whether there is an interaction between Vγ9Vδ2 T cells and aHSCs and (2) whether there is translational potential for using adoptively transferred Vγ9Vδ2 T cells in immunotherapy for the treatment of diseases involving aHSCs.

## Materials and Methods

### Patients and Methods

Blood samples were collected from 33 healthy individuals and 25 cirrhosis patients. Cirrhosis was determined by clinical judgment at the discretion of the treating physicians based on a combination of biochemical parameters, clinical signs, and radiologic and ultrasonic laboratory tests. Age- and sex-matched healthy individuals were enrolled as controls. The study protocol was approved by the Institutional Review Board of Tsinghua University. Written informed consent was obtained from each subject.

### Mice

Rag2^−/−^γc^−/−^ mice were purchased from the Jackson Lab and maintained in an SPF animal facility at the Laboratory Animal Research Center, Tsinghua University. All of the animal experiments were approved by the Institutional Animal Care and Use Committee of Tsinghua University.

### Culture Conditions for LX-2 Cells and Huh 7 Cells

The human LX-2 cell line (purchased from Cancer Research Institute, Xiangya Medical School, Central South University, Changsha) and the Huh 7 cell line (purchased from Institute of Biochemistry and Cell Biology, Chinese Academy of Sciences, Shanghai, China) were cultured in uncoated dish under DMEM containing 10% fetal bovine serum (FBS) in a 5% CO_2_ atmosphere at 37°C.

### Chemical Reagents

BPH-series compounds were synthesized according to reported procedures, and were characterized by ^1^H NMR, ^31^P NMR, and HR-MS. The full characterization of BPH-1236, a representative lipophilic bisphosphonate, is given as follows. ^1^H NMR (400 MHz, D_2_O) δ: 8.45 (s, 1 H), 7.28 (s, 1 H), 7.20 (s, 1 H), 4.45 (t, *J* = 9.6 Hz, 2 H), 4.00 (t, *J* = 7.2 Hz, 2 H), 1.67 (m, 2 H), 1.08 (m, 14 H), 0.62 (t, *J* = 7.2 Hz, 3 H). ^31^P NMR (162 MHz, D_2_O) δ: 15.20.

### Expansion of Vγ9Vδ2 T Cells

Peripheral blood mononuclear cells (PBMCs) were isolated from whole blood samples of healthy donors using a standard Ficoll-Paque gradient centrifugation process. For large-scale cultures, PBMCs were cultured in RPMI 1640 medium supplemented with 10% FBS, 1% Penicillin-Streptomycin, 150 U/mL human rIL-2 (PeproTech), 2 mM l-glutamine, 50 µM β-mercaptoethanol, 1% MEM non-essential amino acids, and 5 µM zoledronate (Zometa; AvaChem Scientific) or 1 µM BPH-1236. The cultures were maintained at a cell density of 2 × 10^6^ cells/mL. For the expansion assay, PBMCs were cultured in 96-well round plates at a density of 1 × 10^5^ cells/well in culture medium with different concentrations of zoledronate or BPH-1236 with/without simvastatin. Fresh medium containing human rIL-2 (150 U/mL), but without test compounds, was added every 2–3 days. Cells were harvested on day 9–14, and the frequency and phenotype of Vγ9Vδ2 T cells were evaluated by flow cytometry. Vγ9Vδ2 T cells (purity >90%) were used in further experiments or were stored in liquid nitrogen. In some experiments, expanded Vγ9Vδ2 T cells were further purified by using Anti-TCR γδ MicroBead Kits (Miltenyi Biotec), according to the manufacturer’s instructions. These experiments were carried out with the donor’s consent and were approved by the Institutional Review Board of Tsinghua University.

### Vγ9Vδ2 T Cells Analysis by Flow Cytometry

The expanded cells were washed and suspended in 100 µL buffer containing PBS (pH 7.2), bovine serum albumin (0.5%), and EDTA (2 mM). Vγ9Vδ2 T cells were labeled using PE or FITC-conjugated anti-human TCR Vδ2 antibody and APC or FITC-conjugated anti-human CD3 antibody (Miltenyi Biotec). For phenotype analysis, the expanded cells were additionally labeled with Vioblue-conjugated anti-human CD27 and PE-conjugated anti-human CD45RA (Miltenyi Biotec). The staining step was done at 4°C for 30 min; samples were washed with 1 mL PBS and were then analyzed with a FACSAria SORP (BD) flow cytometer. Data were analyzed using the FlowJo program.

### Cytotoxicity of Vγ9Vδ2 T Cells against LX-2 Cells

CytoTox 96^®^ Non-Radioactive Cytotoxicity Assay kits (Promega), which are based on the colorimetric detection of the released enzyme lactate dehydrogenase (LDH), was used to determine specific cytotoxicity. Vγ9Vδ2 T cells (Effector, E) were co-cultured with LX-2 cells (Target, T), which were pretreated with N-BPs for 4 h at specific E/T ratios for 4 h (indicated in figure captions). In some cases, after 4 h co-cultured, the number and area of Vγ9Vδ2 T cells clusters were determined using IncuCyte (Essen BioScience) image analysis software. Three different locations per well were imaged with a 10 × objective lens. Clusters were defined as cell aggregates occupying an area at least 300 µm^2^ and were displayed as the number and area of clusters per well. In some experiments, Transwell^®^ units (pore size 0.4 µm, Corning) were used to separate Vγ9Vδ2 T cells from LX-2 cells. In some cases, the neutralization antibodies anti-NKG2D (10 µg/mL, BD), anti-FasL (10 µg/mL, Biolegend), anti-TRAIL (10 µg/mL, Biolegend), anti-TCR γδ (10 µg/mL, Biolegend), and their relevant isotype controls, were individually added in the co-cultures to block the NKG2D-, FasL-, TRAIL-, and TCR γδ- mediated pathways. To block perforin and granzyme B pathways, the perforin inhibitor Concanamycin A (CMA, Selleck) (1 µg/mL) and granzyme B inactivator BCL-2 (1 µg/mL, R&D Systems) were used (15).

### Atomic Force Microscopy Assay

Atomic force microscopy-based single-cell force spectroscopy (AFM-SCFS) was performed with a JPK CellHesion unit as previously described (16). Briefly, LX-2 cells treated with or without 5 µM BPH-1236 were cultured on an uncoated glass substrate that was placed in an AFM-compatible environmental chamber to maintain 37°C and 5% CO_2_. A Cell-Tak (Sigma-Aldrich) coated cantilever was used to glue individual Vγ9Vδ2 T cells and glued cells were used immediately to interact with adherent LX-2 cells. In each approach-retract probing cycle, the AFM cantilever carrying the Vγ9Vδ2 T cell was pressed to create contact with an LX-2 cell with a setpoint of 0.5 nN for a period of 2 s. Then, the cantilever was retracted from the cell of interest until detachment was achieved. The probing process was repeated until a minimum of 10 force curves were collected for each Vγ9Vδ2 T cell-LX-2 cell pair. Five independent pairs of Vγ9Vδ2 T cell-LX-2 cell were tested. The force curves were analyzed using JPK image processing software (17).

### Time-Lapse Confocal Microscopy

5(6)-Carboxyfluorescein *N*-hydroxysuccinimidyl ester (CFSE; an esterase substrate that fluoresces when hydrolyzed, eBioscience) labeled LX-2 cells (green) were pretreated with BPH-1236 (5 μM) for 4 h and then co-incubated with expanded Vγ9Vδ2 T cells labeled with LysoTracker Red (red, Life Technologies) for 1 h. Visualization of perforin/granzyme release at the single-cell level was performed using time-lapse confocal microscopy (Nikon A1Rsi). Data were analyzed using NIS Viewer.

In some cases, Vγ9Vδ2 T cells were co-incubated with LX-2 cells (pretreated with BPH-1236 for 4 h) in the presence of PI (Beyotime, China) and Hoechst 33258 (Life Technologies). Visualization of PI diffusion at the single-cell level was performed using Spinning Disk with FV1200 microscope (PerkinElmer). Images were acquired about every 88 s, with the overlay of the Hoechst 33258 (blue)/PI (red)/Brightfield was shown. Data were analyzed using Volocity (PerkinElmer).

### Construction of LX-2 Cells and Huh 7 Cells Stably Expressing Luciferase

LX-2/luciferase (Luc) cells and Huh 7/Luc cells were established by the stable transfection of the firefly luciferase gene (pPLV-Luc-GFP; kind gift from Professor Yanan Du, Tsinghua University) using linear polyethylenimines (PEI; Polysciences) reagent.

### Huh 7 Cells Proliferation Assay Impacted by LX-2 Cells

Huh 7/Luc cells were cultured in 96 round bottom plates with culture medium or LX-2 conditioned medium (CM) (72 h culture supernatant from LX-2 cells) for 72 h, the number of viable cells was determined by measuring luciferase activity after adding luciferin (China Cellcyto) by using an IVIS imaging system (PerkinElmer).

### Wound Healing Assay

Huh 7/Luc cells were seeded on 96-well plates at 2 × 10^4^ cells per well and incubated overnight. Cells were “wounded” using a cell wound maker (ESSEN Bioscience) and cultured with/without LX-2 cells conditioned medium (CM). To measure cell migration, microscopic photographs were taken at 0 and 48 h after injury using an Operetta CLS High-Content Analysis System (PerkinElmer).

### Vγ9Vδ2 T Cells Homing-In Assays

DiR (1,1′-dioctadecyltetramethyl indotricarbocyanine iodide; GeneCopoeia) labeled Vγ9Vδ2 T cells (10 × 10^6^ per mouse) were *i.v*. injected into Rag2^−/−^γc^−/−^ mice (6–8 weeks old), and the distribution of Vγ9Vδ2 T cells was evaluated by measuring DiR fluorescence using an IVIS imaging system (PerkinElmer).

### LX-2/Luc Cells or Huh 7/Luc Cells Orthotopic Rag2^−/−^γc^−/−^ Mouse Model and Vγ9Vδ2 T Cells Adoptive Therapy

The liver orthotopic model was established using a liver submucosal injection method. Liver submucosal injection accessed by midline laparotomy using aseptic technique under a stereomicroscope. Specifically, 1 × 10^6^ LX-2/Luc cells or 1 × 10^6^ Huh 7/Luc cells (suspended in 20 µL Opti-MEM) were injected into liver submucosal of Rag2^−/−^γc^−/−^ mice of 6–8 weeks age under anesthesia using isoflurane. About 7 days later, when discernible and orthotopic LX-2 cells xenografts (or Huh 7 tumors) formed, mice were divided into groups and treated with Vγ9Vδ2 T cells (10 × 10^6^/mouse, *i.v*.) with or without BPH-1236 (1 mg/kg, *i.v*.) (BPH-1236 was administrated 4 h before Vγ9Vδ2 T cells injection). The treatments were given at day 7 (after transplantation) in LX-2**/**Luc cells orthotopic mice, and at day 7 as well as day 15 in Huh 7**/**Luc cells orthotopic mice. The tumor burden of Huh 7/Luc cells and LX-2/Luc cells xenografts in livers were evaluated by measuring luciferase activity after luciferin injection by using an IVIS imaging system (PerkinElmer). The liver and tumors were harvested and photographed at day 48 after transplantation from Huh 7/Luc cells orthotopic Rag2^−/−^γc^−/−^ mice. The tumor sizes were measured with a digital caliper and tumor volume (cm^3^) was calculated as *V* = long diameter × short diameter^2^ × 0.5.

### Huh 7/Luc Cells and/or LX-2/Luc Cells Spleen Model

1 × 10^6^ Huh 7/Luc cells and/or 1 × 10^6^ LX-2/Luc cells (suspended in 50 µL Opti-MEM) were injected into spleens of 6–8 weeks old Rag2^−/−^γc^−/−^ mice under isoflurane anesthesia. The tumors in livers and spleens were harvested at day 43. Survival of mice was monitored and recorded.

### Statistical Analysis

The two-tailed Student’s t-tests were used for comparing the significance of differences between groups. Statistical analyses and graphing were performed using GraphPad Prism 5 software. All values are presented as mean ± SEM. *P < 0.05; **P < 0.01; ***P < 0.001.

## Results

### Vγ9Vδ2 T Cells Are Reduced in Cirrhosis Patients

**Figure 1:**
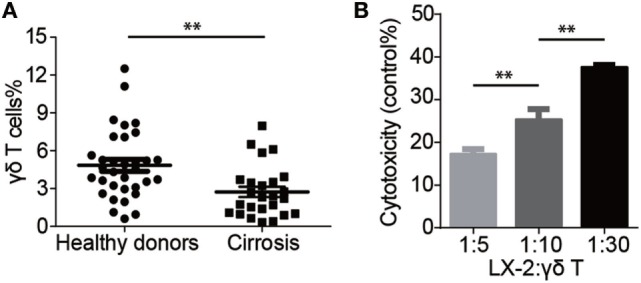
Vγ9Vδ2 T cells are reduced in cirrhosis patients. **(A)** Percentages of Vγ9Vδ2 T cells (γδ T) in peripheral blood mononuclear cells (PBMCs) of cirrhosis patients (n = 25) and sex- and age-matched healthy donors (n = 33). **P < 0.01. **(B)** Cytotoxicity of Vγ9Vδ2 T cells (effector cells, E) against human activated hepatic stellate cell line LX-2 cells (target cells, T) at different ratios. Cytotoxicity was analyzed using the CytoTox 96 Non-Radioactive Cytotoxicity Assay with 1 × 10^4^ LX-2 cells after 4 h co-culture with various amounts of Vγ9Vδ2 T cells. Data are presented as mean ± SEM of three replicates from a representative experiment of two independent experiments. **P < 0.01.

We determined the frequencies of peripheral circulating Vγ9Vδ2 T cells in healthy donors (n = 33) and in cirrhosis patients (n = 25; n = 22 hepatitis B-progressed cirrhosis, n = 3 alcoholic cirrhosis). The number of peripheral Vγ9Vδ2 T cells in cirrhosis patients was reduced by ~1.8 × in cirrhosis patients compared to healthy controls (Figure 1A), suggesting the possibility of a protective role for this subset of T cells in the development of liver cirrhosis. We next tested the hypothesis that Vγ9Vδ2 T cells might be cytotoxic to aHSCs. We used LX-2 cells, a widely accepted human hepatic stellate cell line that is activated when cultured in uncoated plastic dishes and that has been extensively used in studies of the progression of human hepatic fibrogenesis (18, 19). We evaluated cytotoxicity by monitoring the release of an LDH marker (20) and found that aHSCs were killed by *ex vivo*-expanded Vγ9Vδ2 T cells from healthy donors. These Vγ9Vδ2 T cells killed 18% of aHSCs at an effector:target (E:T) ratio of 5:1, and 35% of aHSCs at an E:T ratio of 30:1 (Figure 1B).

### Bone Resorption Drug Zoledronate Enhances the Susceptibility of aHSCs to Vγ9Vδ2 T Cells Killing

**Figure 2:**
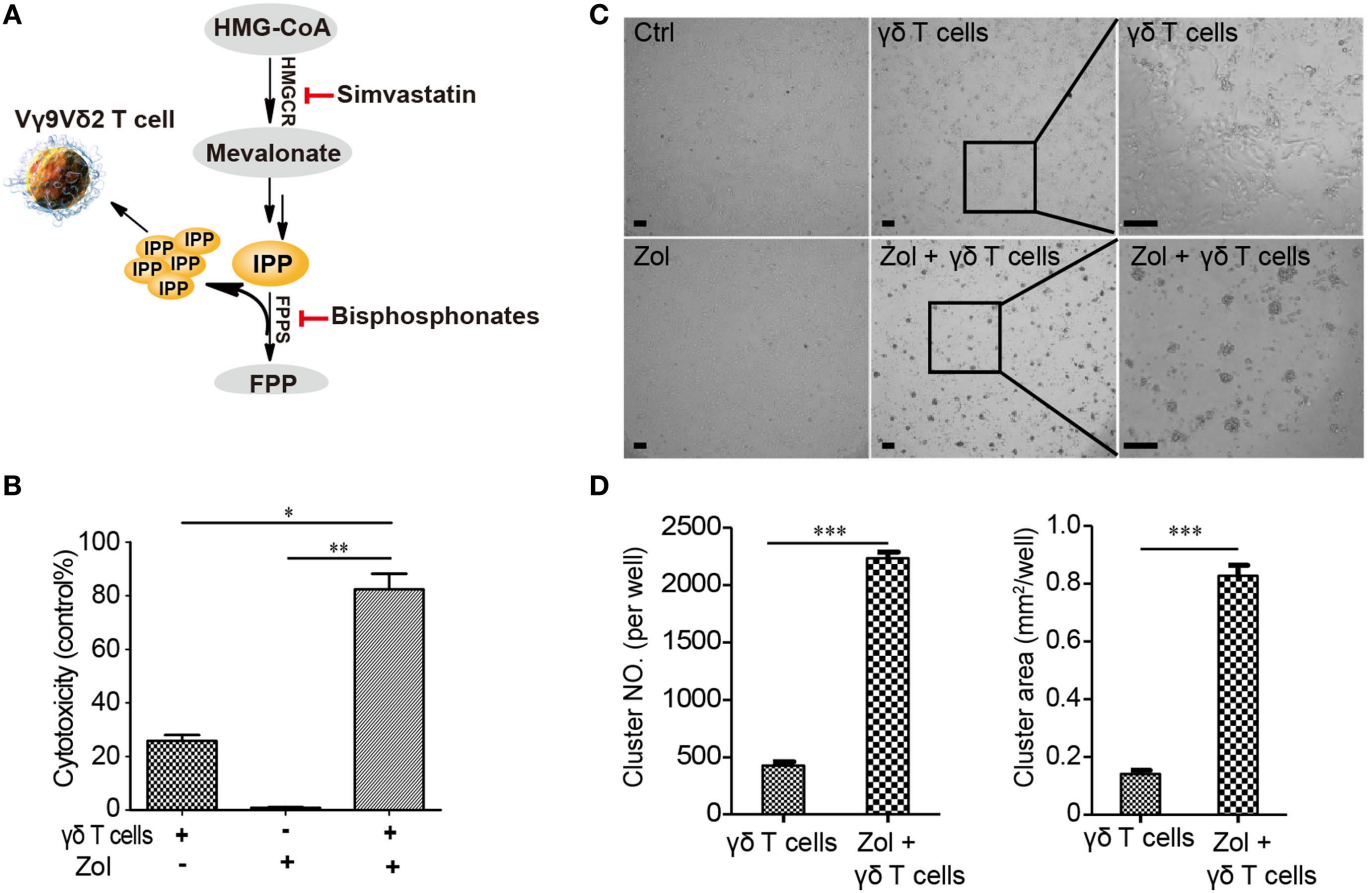
Zoledronate enhances the killing of activated hepatic stellate cells (aHSCs) by Vγ9Vδ2 T cells. **(A)** Schematic illustration of the mevalonate pathway and the function of simvastatin and bisphosphonates (HMGCR, 3-hydroxy-3-methylglutaryl-CoA reductase; FPPS, farnesyl pyrophosphate synthase; IPP, isopentenyl pyrophosphate). **(B)** LX-2 cells (T) pretreated with 5 µM zoledronate (bisphosphonate) were more sensitive than untreated controls to killing by Vγ9Vδ2 T cells (E) at an E:T ratio of 10; recording of lactate dehydrogenase (LDH) activity. Data are presented as mean ± SEM of three replicates from a representative experiment of three independent experiments. **(C)** Representative transmitted-light images showing LX-2 cells pretreated with zoledronate (5 µM) resulted in more killing and more clustered Vγ9Vδ2 T cells. Scale bar, 100 µm. **(D)** The number (NO.) and size (area) of Vγ9Vδ2 T cells clusters in **(C)**. Data are presented as mean ± SEM of four replicates from a representative experiment of two independent experiments. *P < 0.05; **P < 0.01, ***P < 0.001.

Vγ9Vδ2 T cells are currently being used in adoptive immunotherapies to target a broad range of cancers including leukemia (21), melanoma (22), colon carcinoma (23), and breast cancer (24), among others (25–27). Vγ9Vδ2 T cells recognize tumor cells by sensing their increased accumulation of the phosphorylated metabolite isopentenyl pyrophosphate (IPP). We speculated that activated Vγ9Vδ2 T cells might be able to recognize aHSCs in a similar manner. Biochemically, IPP is a substrate of the farnesyl diphosphate synthase (FPPS) enzyme, and inhibition of FPPS by drugs such as nitrogen-containing bisphosphonates is known to result in elevated IPP levels (Figure 2A). IPP can bind to and induce a conformational change in the transmembrane protein butyrophilin 3 A1 (BTN3A1) (28–32), which is recognized by the Vγ9Vδ2 T cell receptor, ultimately resulting in target cell killing. In theory, assuming that a similar mechanism is involved in the killing of aHSCs by Vγ9Vδ2 T cells, the increase in IPP levels resulting from bisphosphonate treatment should increase the susceptibility of aHSCs to cytolysis by Vγ9Vδ2 T cells.

We used zoledronate, a potent bisphosphonate drug in current clinical use (33), to test this hypothesis by monitoring the ability of Vγ9Vδ2 T cells to kill zoledronate-treated aHSCs (LX-2 cells). At an E:T ratio of 10:1, LX-2 cells pretreated with 5 µM zoledronate were far more susceptible to killing by Vγ9Vδ2 T cells than were untreated control cells, and zoledronate alone had essentially no effect on LX-2 cells viability (Figure 2B). Consistently, zoledronate-pretreated cells were more extensively surrounded by clustered Vγ9Vδ2 T cells than were the untreated cells (Figures 2C,D). Together, these results show that human Vγ9Vδ2 T cells can kill aHSCs and demonstrate that the efficiency of this killing can be enhanced by treating aHSCs with the bisphosphonate drug, zoledronate.

### BPH-1236, a Lipophilic Analog of Zoledronate, Performs Better and Functions via Inhibiting FPPS

Zoledronate and other bisphosphonates were initially developed for the treatment of bone diseases such as osteoporosis, Paget’s disease, and hypercalcemia due to malignancy. Chemically, they are extremely polar, and are rapidly removed from blood circulation via binding to bone (34). While these features are desirable for bone-targeting drugs, they represent challenges for their use in other indications. For example, the poor pharmacokinetic properties of zoledronate (bone affinity and short circulation) are associated with risks of osteonecrosis of the jaw and renal impairment. In previous work, we generated a new class of drug leads called lipophilic bisphosphonates for the treatment of both KRAS-driven lung adenocarcinomas (35) and malaria (36). These compounds have almost no affinity for bone (37), and remain in circulation much longer than do conventional bisphosphonates (35). Building from our discovery that zoledronate can enhance the killing of aHSCs by Vγ9Vδ2 T cells, we next explored the possibility that the lipophilic bisphosphonates might have translational potential as lead compounds for treating liver disease.

**Figure 3:**
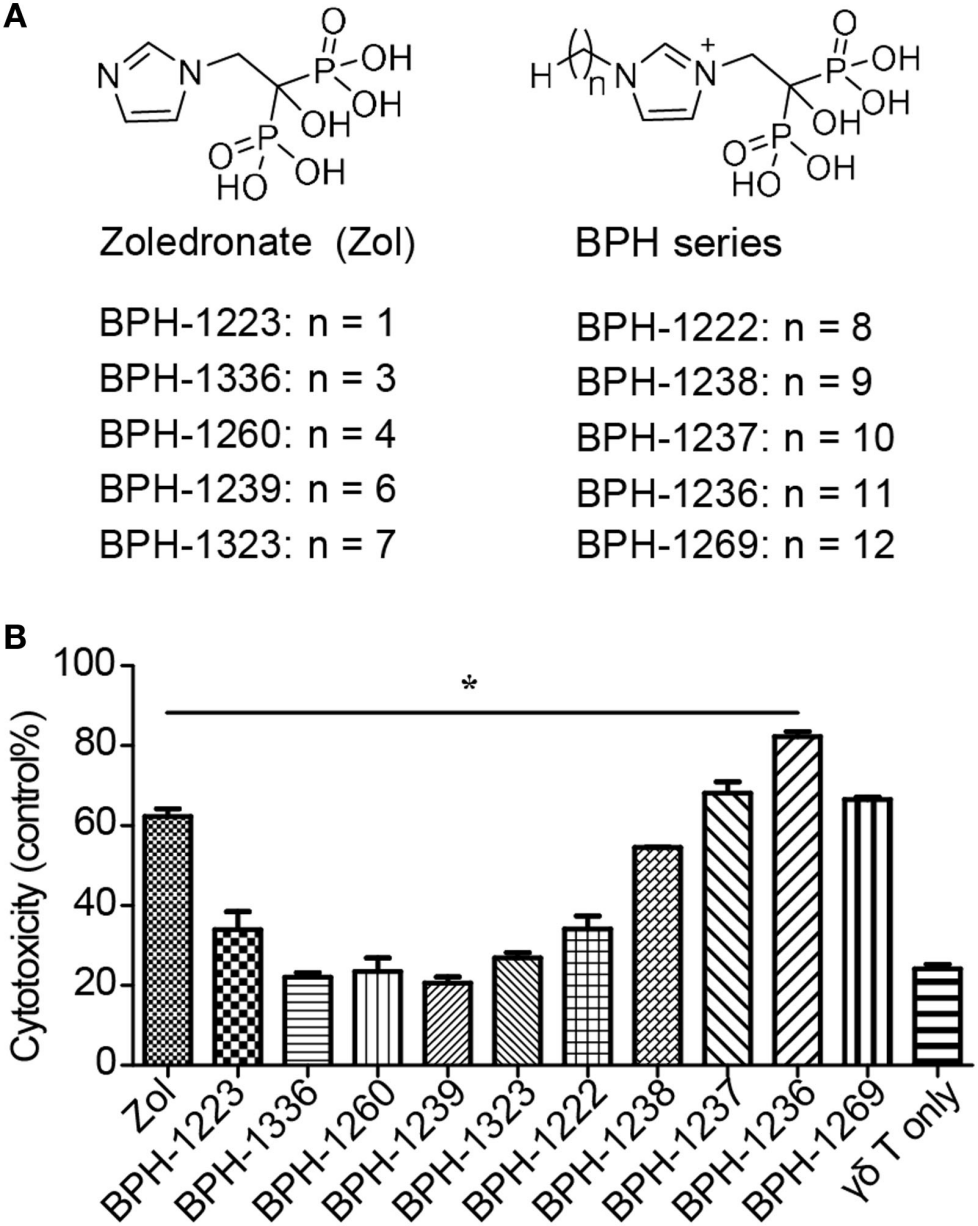
BPH-1236, a lipophilic analog of zoledronate, performs the better killing of activated hepatic stellate cells (aHSCs) by Vγ9Vδ2 T cells. **(A)** Chemical structures of zoledronate and its lipophilic analogs (BPH series). **(B)** Specific lysis of LX-2 cells (T) pretreated with various lipophilic BPH series (5 µM) by Vγ9Vδ2 T cells (E) at an E:T ratio of 10; recording of lactate dehydrogenase (LDH) activity. Data are presented as mean ± SEM of three replicates from a representative experiment of two independent experiments. *P < 0.05.

We tested the ability of 10 lipophilic analogs of zoledronate (structures shown in Figure 3A) with various alkyl chain lengths to potentiate the effects of Vγ9Vδ2 T cells killing of aHSCs (LX-2 cells). These alkyl chains do not interfere with the enzyme binding but increase the lipophilicity of the molecule. Most of these bisphosphonates are potent FPPS inhibitors (35) and as expected they increased the susceptibility of aHSCs to cytolysis by Vγ9Vδ2 T cells, and BPH-1236 was the most potent compound tested (Figure 3B). Then we ask the question whether BPH-1236 functions by increasing the levels of IPP, a danger signal recognized by Vγ9Vδ2 T cells.

**Figure 4:**
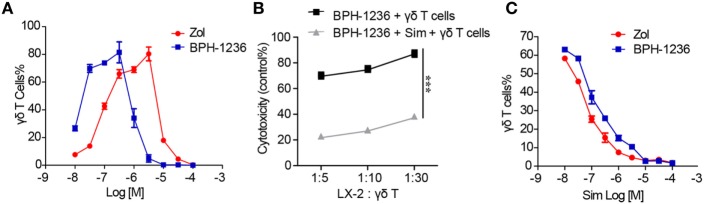
BPH-1236 performs better and functions via inhibiting farnesyl diphosphate synthase (FPPS). **(A)** Response of human blood Vγ9Vδ2 T cells to zoledronate or BPH-1236 treatment. Isolated human peripheral blood mononuclear cells (PBMCs) were treated with zoledronate or BPH-1236 for 3 days, and cells were allowed to proliferate for another 9 days, followed by staining for CD3 and TCR Vδ2. **(B)** The rescue effect of simvastatin on the cytotoxicity of Vγ9Vδ2T cells against LX-2 cells that were pretreated with BPH-1236. ***P < 0.001. **(C)** The rescue effect of simvastatin on the response of human blood Vγ9Vδ2 T cells to zoledronate or BPH-1236. All data are presented as mean ± SEM of three replicates from a representative experiment of three independent experiments.

In preclinical or clinical settings, zoledronate stimulates PBMCs to expand Vγ9Vδ2 T cells by increasing the IPP levels inside PBMC. We found that pulsing BPH-1236 onto PBMC also efficiently stimulated a major expansion of the number of Vγ9Vδ2 T cells, with a 10-fold lower EC_50_ than did zoledronate (Figure 4A), and an increased population of effector memory cells (Figure S1 in Supplementary Material). The dual biological effects of BPH-1236 (sensitizing aHSCs to Vγ9Vδ2 T cells killing and *ex vivo* Vγ9Vδ2 T cells stimulation) are consistent with its FPPS inhibitor function in increasing IPP levels. To further confirm this, we used simvastatin, an HMG-CoA reductase inhibitor that inhibits IPP production, to rescue these effects. As expected, treatment with a combination of BPH-1236 plus simvastatin greatly diminished aHSCs killing (Figure 4B) and *ex vivo* Vγ9Vδ2 T cells stimulation by BPH-1236 (Figure 4C). Thus, clearly, BPH-1236 functions by increasing IPP levels in aHSCs, making them more susceptible to Vγ9Vδ2 T cells recognition and killing.

### Cytotoxicity Is Mediated by Direct Cell-to-Cell Contact, with BPH-1236 Increasing the Adhesion between aHSCs and Vγ9Vδ2 T Cells

**Figure 5:**
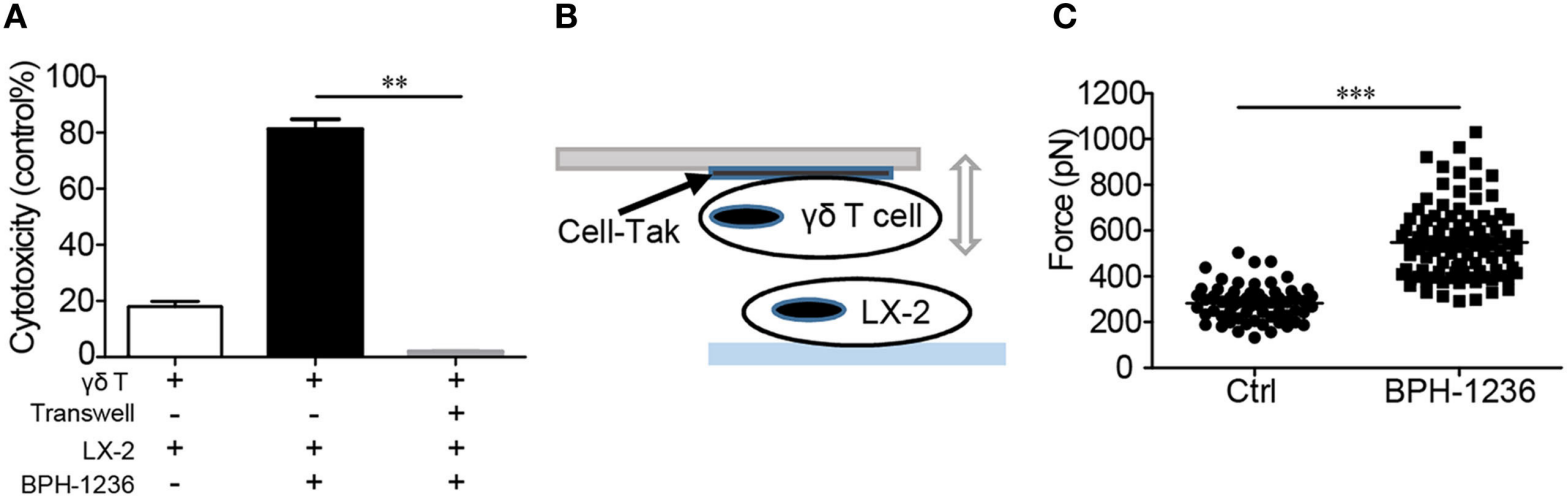
Cytotoxicity is mediated by direct cell-to-cell contact, with BPH-1236 increasing the adhesion between activated hepatic stellate cells (aHSCs) and Vγ9Vδ2 T cells. **(A)** The Vγ9Vδ2 T cells were directly co-cultured with LX-2 cells or by using a Transwell system (at right). Specific lysis of LX-2 cells was recorded. Data are presented as mean ± SEM of three replicates from a representative experiment of three independent experiments. **P < 0.01. **(B)** A schematic diagram for the atomic force microscopy-based single-cell force spectroscopy (AFM-SCFS) assay setup. Cell-Tak = cell adhesive. **(C)** Adhesion atomic forces between individual Vγ9Vδ2 T cells and LX-2 cells treated with or without BPH-1236 (5 µM), measured by AFM. All data points are from five independent Vγ9Vδ2 T cell/LX-2 cell pairs for each condition that were collected on the same day. Each group contains at least 50 data points from five pairs of cells with 10 cycles. ***P < 0.001.

We next sought to investigate in more detail the cell–cell recognition and killing of aHSCs by Vγ9Vδ2 T cells. It is reported that IPP inside the target cell will bind to the intracellular part of BTN3A1, inducing an extra-cellular conformation change that is recognized by TCR γδ (28–32). We deduced that a cell-to-cell contact is needed between aHSCs and Vγ9Vδ2 T cells for recognition and cytolysis. Using a Transwell^®^ apparatus, we found that killing of LX-2 cells by Vγ9Vδ2 T cells occurred only under cell-to-cell contact conditions (Figure 5A). To quantify this cell–cell interaction, we used a technology called AFM-SCFS, a method that enables direct measurement of the adhesion force between individual pairs of interacting cells *in vitro* (Figure 5B) (16, 38, 39). We glued Vγ9Vδ2 T cells to the tip of a flat cantilever and used it to approach LX-2 cells placed on a glass substrate. The binding forces were measured using a cyclical approach-retract method. In the retraction phase, an average force of 280 ± 10 piconewtons was required for complete detachment (Figure 5C). However, pretreatment of the LX-2 cells with BPH-1236 increased the force (Figure 5C; Figure S2A in Supplementary Material) or the work (Figures S2A,B in Supplementary Material) required to detach cells by a factor of two. This BPH-1236 mediated increase in the adhesion strength between LX-2 cell and Vγ9Vδ2 T cell is consistent with our observation that BPH-1236 treatment enhances the ability of Vγ9Vδ2 T cells to kill aHSCs.

### The Cytolytic Process Is Largely Dependent on the Perforin/Granzyme B Pathway

**Figure 6:**
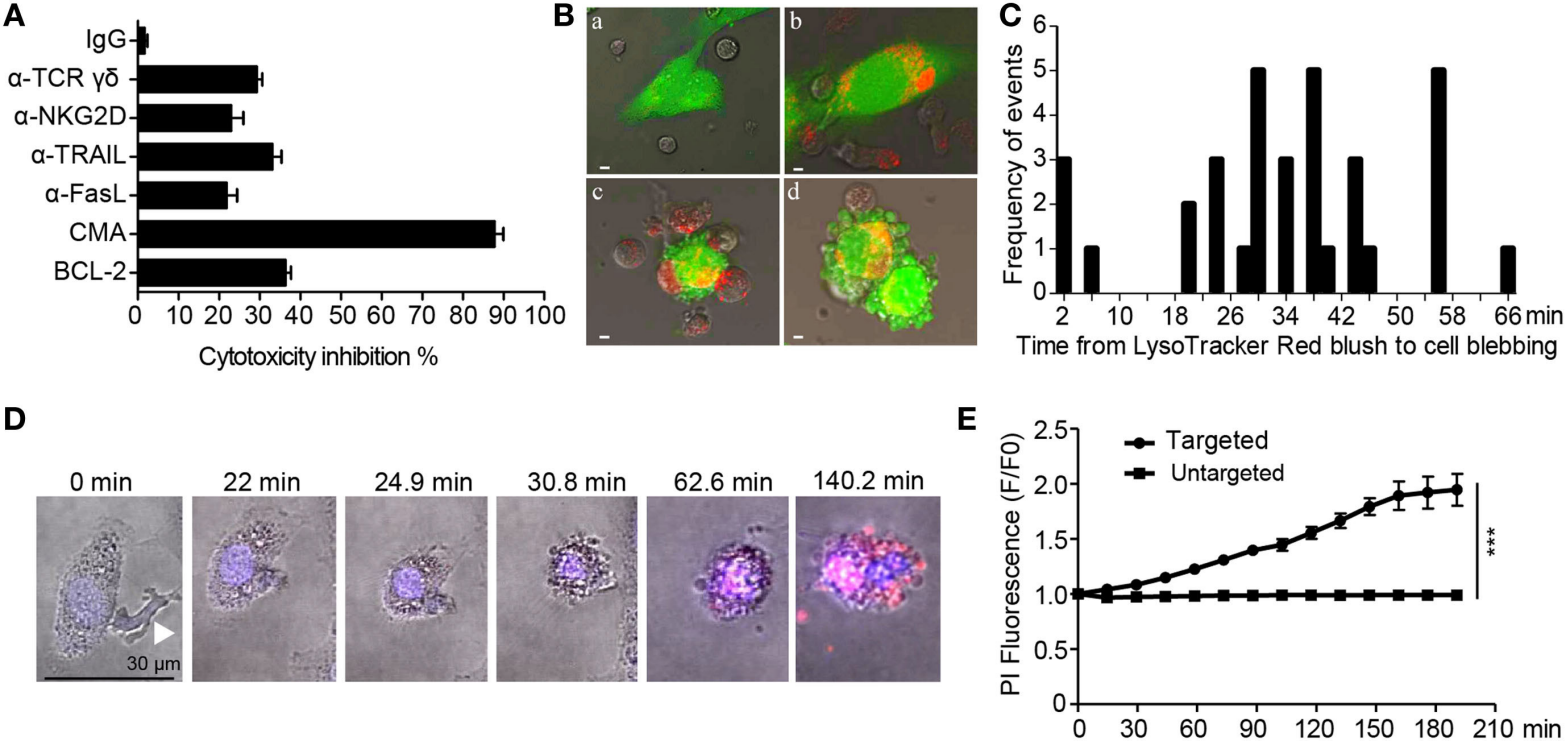
The cytolytic process is largely dependent on the perforin/granzyme B pathway. **(A)** Specific lysis of LX-2 cells (pretreated with 5 µM BPH-1236) by expanded Vγ9Vδ2 T cells in the presence of neutralization antibodies or inhibitors: anti-NKG2D (α-NKG2D), anti-FasL (α-FasL), anti-TRAIL (α-TRAIL), anti-TCR γδ (α-TCR γδ), the perforin inhibitor Concanamycin A (CMA), granzyme B inhibitor (BCL-2). Data are presented as mean ± SEM of three replicates from a representative experiment of three independent experiments. **(B)** Confocal microscopy of the co-culture of LX-2 cells (5(6)-carboxyfluorescein *N*-hydroxysuccinimidyl ester (CFSE) labeled, green, pretreated with 5 µM BPH-1236 for 4 h) and Vγ9Vδ2 T cells (labeled with LysoTracker Red). a, Vγ9Vδ2 T cells were seeded along with BPH-1236 pretreated LX-2 cell cultures; b, Vγ9Vδ2 T cells moved toward the LX-2 cells, and released perforin/granzyme lytic granules through the immunological synapse into LX-2 cells; c, the target LX-2 cell lost its initial shape and started to acquire a round morphology; d, LX-2 cells broke up into many membrane-bound bodies, with apoptosis. Scale bar, 5 µm. **(C)** Frequency distribution of the times (minutes) between the LysoTracker Red blush to the first sign of cell blebbing. Columns represent 2-min time intervals (n = 34 apoptotic target cell deaths). **(D)** Time-lapse microscopy of Vγ9Vδ2 T cells and BPH-1236 pretreated LX-2 cells in the presence of PI and Hoechst 33258. Images were acquired about every 88 s and show the Hoechst 33258 (blue)/PI (red)/Brightfield overlay. White arrow indicates the Vγ9Vδ2 T cell. Images depict cytosolic diffusion of PI into the target LX-2 cells from the point of Vγ9Vδ2 T cell contact with LX-2 cell (0 min), followed by cell rounding, membrane blebbing, and late stage of PI dispersion. Scale bar, 30 µm. Images are representatives of 14 target cells. **(E)** Monitoring the PI fluorescence change over time in targeted (Vγ9Vδ2 T cell hit LX-2 cells) and untargeted (non-hit LX-2 cells). Images were acquired every 88 s. Data represent the mean ± SEM for fold change (F/F0) in PI fluorescence over time (n = 14 Vγ9Vδ2 T cell hit LX-2 cells and n = 12 non-hit LX-2 cells). ***P < 0.001.

We next investigated the signaling cascades evoked by aHSCs-Vγ9Vδ2 T cells recognition. Vγ9Vδ2 T cells kill cancerous cells in a process that involves the TCR γδ and NKG2D receptors, with engagement of Fas-FasL and TRAIL-DR5 (40). Here, we used antibodies to separately block the TCR γδ or NKG2D receptors. This reduced the susceptibility of aHSCs to killing by Vγ9Vδ2 T cells by 30% (a-TCR γδ) and 22% (a-NKG2D) (Figure 6A). Similar modest reductions in susceptibility were observed when we blocked FasL (20%) or TRAIL (33%). However, by far the largest effect on cytolytic activity (~87% inhibition) was observed following the addition of the V-H^+^-ATPase inhibitor, CMA. This compound blocks release of perforin, and hence inhibits perforin/granzyme B lytic activity, although addition of the granzyme B inhibitor BCL-2 alone had a smaller effect (34% inhibition) (Figure 6A).

To visualize the effects of the perforin and granzyme proteins in the killing of BPH-1236 pretreated LX-2 cells by Vγ9Vδ2 T cells, we used LysoTracker Red to trace acidic lytic granules (red) in Vγ9Vδ2 T cells, and CFSE (green) to monitor LX-2 cells. Time-lapse confocal microscopy revealed that lytic granules rapidly converged to the Vγ9Vδ2 T cell/LX-2 cell immune synapse. These granules were then released into the LX-2 cells, resulting in their lysis into many membrane-bound bodies, and cell apoptosis (Figures 6B,C; Video S1 in Supplementary Material). Consistent with the target cell blebbing and lysis, we observed an increase in PI fluorescence over time in the Vγ9Vδ2 T cells hit LX-2 cells when compared with non-hit LX-2 cells (Figures 6D,E). Thus, aHSCs that are pretreated with the lipophilic bisphosphonate BPH-1236 are killed primarily by perforin/granzyme lytic granules released from Vγ9Vδ2 T cells.

### Adoptively Transferred Vγ9Vδ2 T Cells and BPH-1236 Combine to Kill Activated Stellate Cells in an Orthotopic Rag2^−/−^γc^−/−^ Mouse Model

**Figure 7:**
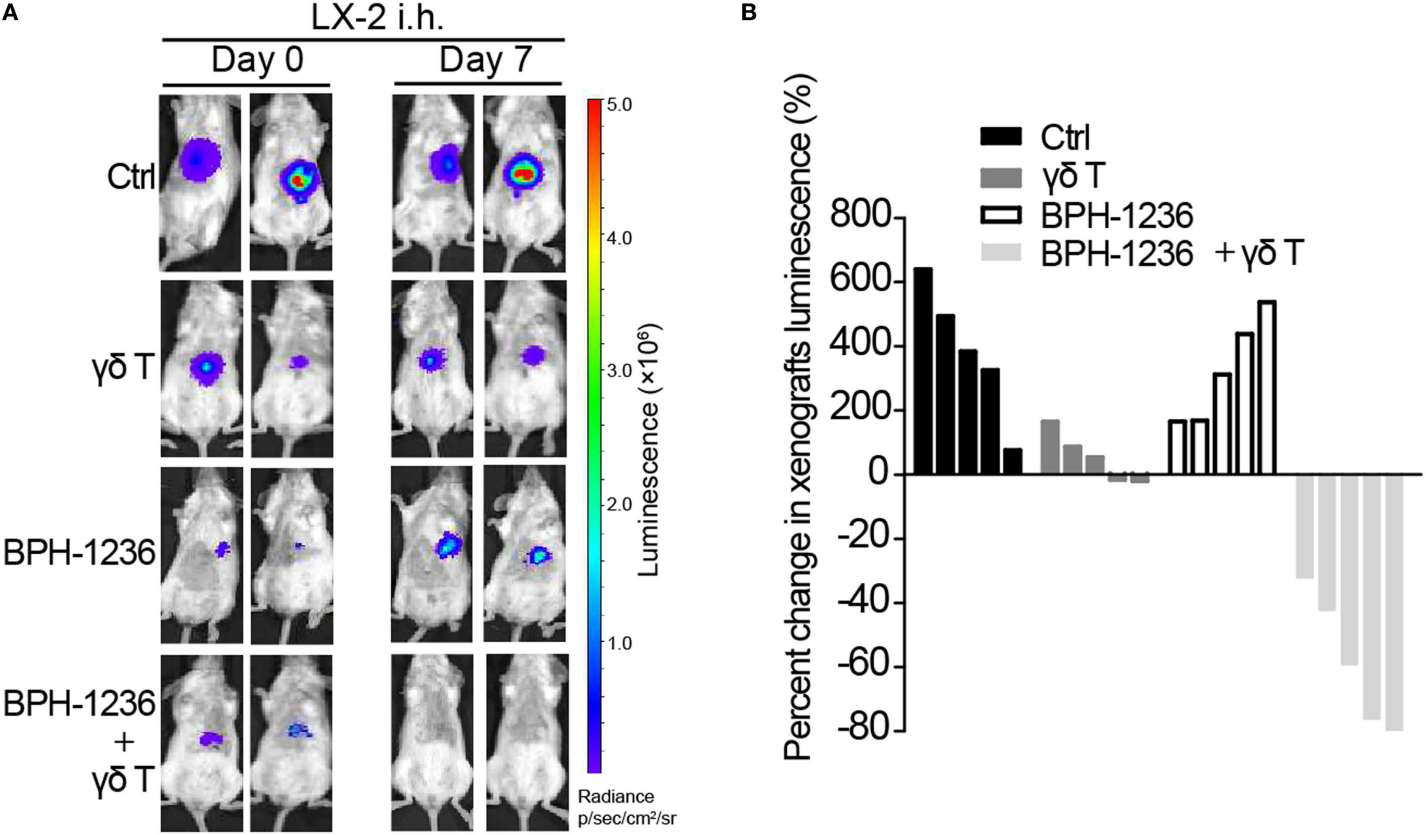
Adoptively transferred Vγ9Vδ2 T cells and BPH-1236 combine to kill activated stellate cells in an orthotopic Rag2^−/−^γc^−/−^ mouse model. **(A)** Representative bioluminescence images showing orthotopic LX-2/Luc cells in Rag2^−/−^γc^−/−^ mice on day 0 (before treatment) and day 7 (7 days after treatment), n = 5 per group. **(B)** Percent changes in LX-2 cells xenografts volume (luminescence value) in (A) from day 0 (baseline) to day 7 are shown for each mouse (n = 5 per group) as a waterfall plot (in comparison with control (Ctrl), γδ T cells: *P* < 0.01; BPH-1236: ns; BPH-1236 + γδ T cells: *P* < 0.05; two-tailed Student’s t-tests).

We then investigated the translational potential of Vγ9Vδ2 T cells for the treatment of aHSCs-mediated diseases. Vγ9Vδ2 T cells target molecules or cell types that are only found in humans and other primates (41). Specifically, stellate cells from mouse liver lack the BTN3A1 gene and are not recognized by Vγ9Vδ2 T cells (41), thus the direct anti-fibrotic efficacy of Vγ9Vδ2 T cells can’t be evaluated in conventional mouse models. We thus chose to test whether Vγ9Vδ2 T cells can kill human aHSCs in immunodeficient mice. It is known that the liver is one of the main organs in which the “homing-in” of adoptively transferred Vγ9Vδ2 T cells occurs (42), implying that liver diseases may be amenable to treatment with Vγ9Vδ2 T cells. In a Rag2^−/−^γc^−/−^ immune-deficient mouse model (15), we observed that *ex vivo*-expanded Vγ9Vδ2 T cells (tail vein injection, stained with a non-diffusing cytoplasmic membrane probe XenoLight DiR) trafficked predominantly to mice livers (Figure S3 in the Supplementary Material). We next assessed the *in vivo* cytotoxicity of Vγ9Vδ2 T cells against aHSCs in an orthotopic mouse model in which LX-2/Luc cells (luciferase-tagged LX-2 cells) were injected into the *tunica serosa* of the livers of Rag2^−/−^γc^−/−^ mice. One week after injection, mice were treated with BPH-1236 (1 mg/kg), followed by the adoptive transfer of 1 × 10^7^ Vγ9Vδ2 T cells (>90% purity). BPH-1236 treatment greatly enhanced the *in vivo* killing efficacy of Vγ9Vδ2 T cells against aHSCs (Figures 7A,B). Our results with this orthotopic model thus clearly suggested the potential for using Vγ9Vδ2 T cells in combination with a lipophilic bisphosphonate to treat aHSCs driving liver diseases (e.g., liver fibrosis, cirrhosis, and even HCC).

### Adoptively Transferred Vγ9Vδ2 T Cells and BPH-1236 Combine to Kill HCC Tumors in an Orthotopic Rag2^−/−^γc^−/−^ Mouse Model

**Figure 8:**
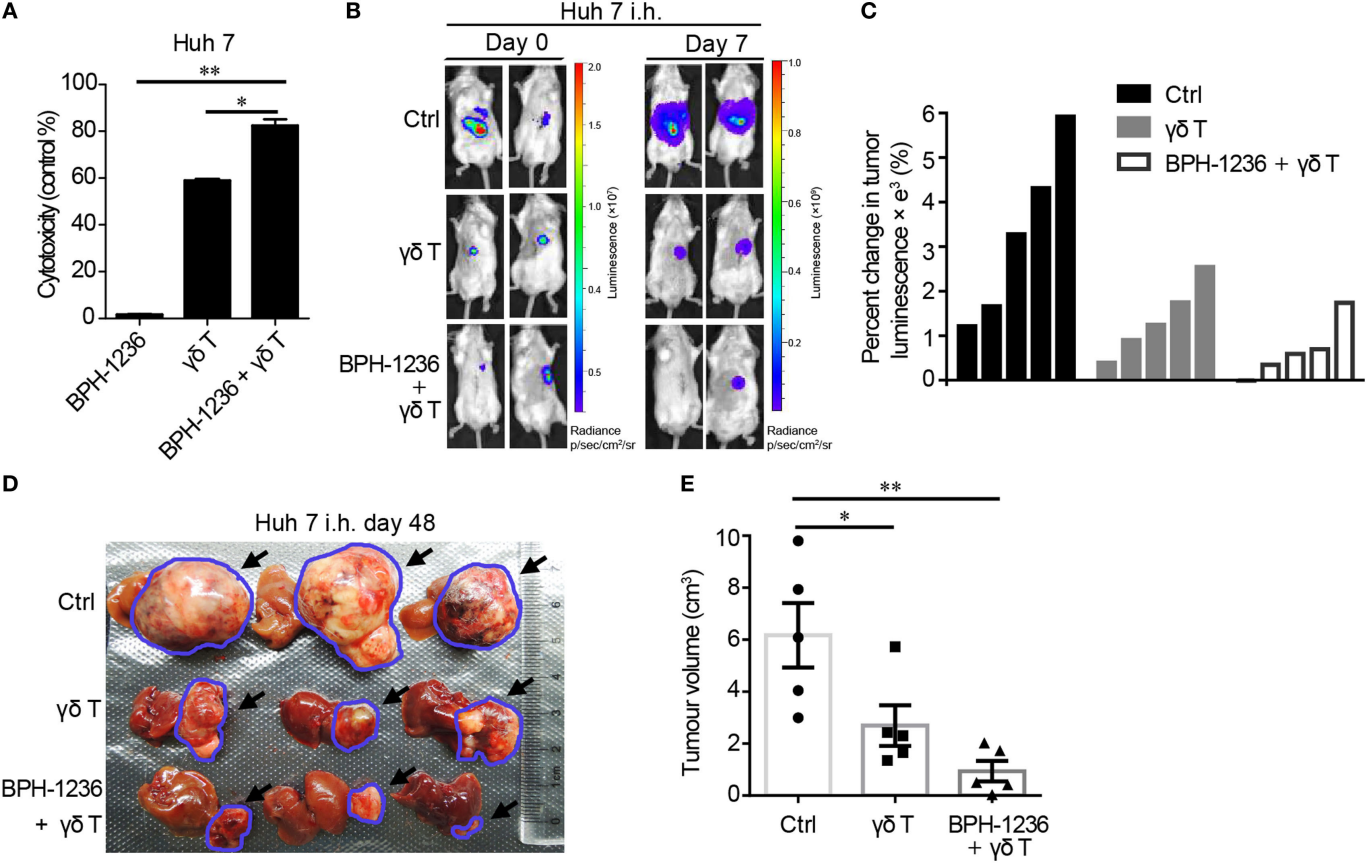
Adoptively transferred Vγ9Vδ2 T cells and BPH-1236 combine to kill hepatocellular carcinoma (HCC) tumors in an orthotopic Rag2^−/−^γc^−/−^ mouse model. **(A)** Cytotoxicity (lactate dehydrogenase (LDH) assay) of Vγ9Vδ2 T cells against human Huh 7 cells pretreated with BPH-1236. Data are presented as mean ± SEM of three replicates from a representative experiment of three independent experiments. **(B)** Representative bioluminescence images showing volume of orthotopic Huh 7/Luc tumors in Rag2^−/−^γc^−/−^ mice on day 0 (before treatment) and day 7 (7 days after treatment); n = 5 per group. **(C)** Percent changes in tumor volume (luminescence value) in (B) from day 0 (baseline) to day 7 are shown for each mouse (n = 5 per group) as a waterfall plot (in comparison with control (Ctrl), γδ T cells: *P* < 0.05; BPH-1236 + γδ T cells: *P* < 0.05; two-tailed Student’s t-tests). **(D)** Representative liver/tumor images from each group (n = 5 per group) at day 48 after tumor transplantation (The treatments were given at day 7 as well as day 15 after Huh 7**/**Luc cells transplantation). Black arrows and blue circles highlight the tumors in liver. **(E)** Tumor volume in (D) at day 48 after tumor transplantation. *P < 0.05; **P < 0.01.

Activated hepatic stellate cells have been reported to promote HCC tumorigenicity (43). We also found that LX-2 cells (aHSCs) conditioned medium (CM) (72 h culture supernatant from LX-2 cells) stimulated human Huh 7 cell (an HCC cell line) growth *in vitro* and increased Huh 7 cell migration (Figures S4A,B in Supplementary Material). We then used an intra-splenic injection model (*in vivo*) and found that Rag2^−/−^γc^−/−^ mice co-injected with Huh 7 cells and LX-2 cells developed more severe liver metastases than did the Huh 7 cells-alone control group, in addition to having lower survival rates (Figures S4C,D in Supplementary Material). We found that BPH-1236 enhanced the killing effect of Vγ9Vδ2 T cells against Huh 7 cells *in vitro* (Figure 8A), as seen with aHSCs. This is not unexpected since cancerous cells have been reported as the main target cells of Vγ9Vδ2 T cells (27). The combination of *ex vivo* expanded Vγ9Vδ2 T cells with BPH-1236 efficiently also shrunk orthotopic HCC tumor burden in Rag2^−/−^γc^−/−^ mice (Figures 8B–E). It is thus clear that Vγ9Vδ2 T cell adoptive transfer with a lipophilic bisphosphonate holds promise as a strategy for fibrosis–cirrhosis associated HCC treatment, since such a strategy killed not only aHSCs (Figure 7), the driving force of HCC, but also tumors (Figure 8) directly.

## Discussion

The results we have described above have both fundamental and translational implications. Both immune cells and aHSCs are important mediators of hepatic fibrosis and their interactions have emerged as important determinants of liver fibrosis progression. The functions of many immune cells toward aHSCs have been studied, and comprehensive understanding of their interaction may lead to novel therapeutic strategies for chronic liver diseases. It has been reported, for example, that natural killer cells can attenuate liver fibrosis *via* killing of aHSCs (7), we showed that individuals with liver cirrhosis have decreased levels of Vγ9Vδ2 T cells. Further, we found that Vγ9Vδ2 T cells kill LX-2 cells (the standard cell line recapitulating most key features of the activated human HSC). These are important observations, implying that Vγ9Vδ2 T cells function as immune surveillance in the pathogenesis of liver diseases. Low Vγ9Vδ2 T cell levels result in less aHSCs killing, and thus enhance the progress of liver damage. On the other hand, these observations suggest an immunotherapeutic strategy for the treatment of liver diseases driven by aHSCs, by using adoptively transferred Vγ9Vδ2 T cells.

**Figure 9:**
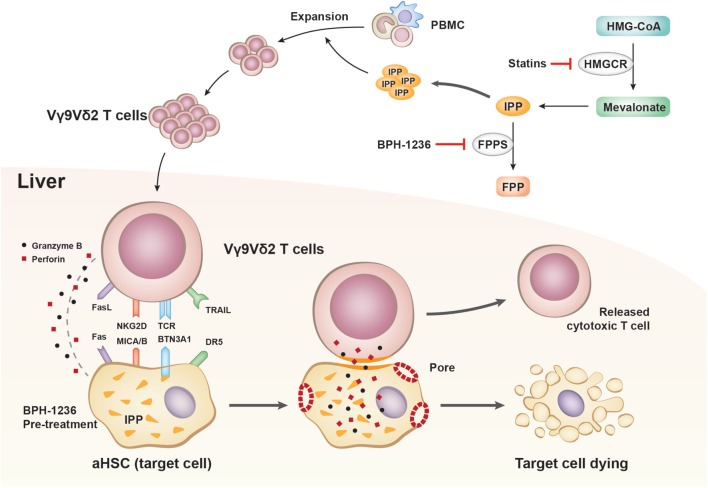
Outlined mechanism underlying the role of Vγ9Vδ2 T cells on activated hepatic stellate cells (aHSCs) apoptosis.

Vγ9Vδ2 T cells respond to non-peptide phosphoantigens in a way not restricted to MHC molecules, thus representing a radical departure from the classical T cell recognition paradigm. For example, Vγ9Vδ2 T cells coordinate an immune response against cancerous cells by sensing the elevated level of IPP produced due to the dysregulation of the mevalonate pathway. In our study, how Vγ9Vδ2 T cells recognize and kill aHSCs is a fundamental question to be answered. Here we observed that bisphosphonates greatly enhanced the killing of aHSCs by Vγ9Vδ2 T cells. It is known that bisphosphonates function by inhibiting FPPS, resulting in IPP accumulation and thus Vγ9Vδ2 T cell recognition. We further found simvastatin, which depletes IPP production, abolished the killing efficacy evoked by bisphosphonates. Undoubtedly, Vγ9Vδ2 T cells sense the IPP levels inside aHSCs. By using AFM-SCFS, we could precisely measure the adhesion force between Vγ9Vδ2 T cell and aHSCs. This technology allowed us, for the first time, to show that a bisphosphonate dramatically increases the adhesion force between a Vγ9Vδ2 T cell and its target cell, thus facilitating perforin/granzyme B lytic activity and target cell death (mechanism outlined in Figure 9).

More than 30 years of Vγ9Vδ2 T cell research have established that these cells play a key role in a host’s response to infections, and some cancers (27). The results presented here add liver ailments to the list of diseases that can be targeted by Vγ9Vδ2 T cells, and open up new possibilities for treating liver fibrosis–cirrhosis–carcinoma using lipophilic bisphosphonate/Vγ9Vδ2 T cell immunotherapies, via targeting aHSCs.

## Ethics Statement

This study was carried out in accordance with the recommendations of “Animal Care and Use of Tsinghua University”. The protocol was approved by the “Institutional Animal Care and Use Committee of Tsinghua University”. This study was carried out in accordance with the recommendations of “Institutional Review Board of Tsinghua University” with written informed consent from all subjects. All subjects gave written informed consent in accordance with the Declaration of Helsinki. The protocol was approved by the “Institutional Review Board of Tsinghua University”.

## Author Contributions

XYZ carried out most experiments, analyzed data; XGZ, YG, and GZ performed clinical blood samples analysis; HX carried out animal experiments; YX synthesized the compounds; YS and NK performed atomic force microscopy assays; XYZ, EO, XGZ, and YZ wrote the manuscript; YZ and XH designed research and edited the manuscript.

## Conflict of Interest Statement

The authors declare that the research was conducted in the absence of any commercial or financial relationships that could be construed as a potential conflict of interest.

## Supplementary Material

Figure S1 Effector memory phenotype of Vγ9Vδ2 T cells stimulated by zoledronate or BPH-1236. Vγ9Vδ2 T cells were assessed by flow cytometric staining for TCR Vδ2, CD45RA and CD27. Flow plots in all panels are representative of at least three independent experiments.

Figure S2 Atomic force microscopy (AFM) force curve assay. **(A)** Two representative force curves showing maximum adhesion forces (blue circles) between individual Vγ9Vδ2 T cell and LX-2 cell untreated (left) or treated (right) with BPH-1236 and the corresponding work (shaded area) required for full separation between the two cells. **(B)** Histograms of work between the two cells with or without BPH-1236 treatment. Each group contains at least 50 data points from five pairs of cells with 10 cycles. ****P* < 0.001.

Figure S3 Homing behavior of Vγ9Vδ2 T cells in Rag2^−/−^γc^−/−^ mice. Vγ9Vδ2 T cells were labeled with XenoLight DiR, and monitored with the IVIS imaging system on day 1, 3, 5 after *i.v*. adoptive transfer (n = 3). One of the two independent experiments is shown.

Figure S4 Activated HSCs promote the growth, migration, and metastasis of human liver cancer cells. **(A)** The growth of Huh 7/Luc cells under the control medium or LX-2 cells conditioned medium (CM) for 72 h, as determined by IVIS imaging. Data are presented as mean ± SEM of four replicates from a representative experiment of two independent experiments. **P* < 0.05. **(B)** Representative micrograph of the areas between scratch fronts after 48 h. The scratched Huh 7/Luc cells were treated with the control medium or LX-2 cells condition medium for 48 h. Data are presented of six replicates from a representative experiment of two independent experiments. **(C)** Representative images of liver metastasis in Huh 7/LX-2 cells or Huh 7 cells spleen xenografts (n = 5 per group). 1 × 10^6^ Huh 7 cells and 5 × 10^5^ LX-2 cells or 1 × 10^6^ Huh 7 were injected into spleen of Rag2^−/−^γc^−/−^ mice at day 0, and livers/tumors were harvested at day 43. Black arrows and blue circles highlight the tumors in liver. **(D)** Survival rate in Huh 7/LX-2 cells or Huh 7 cells spleen xenograft mice; n = 5 per group.

Video S1 Time lapse movie of the interaction between CFSE (green) labeled LX-2 cells and LysoTracker Red (red) labeled Vγ9Vδ2 T cells. LX-2 cells were pretreated with 5 µM BPH-1236 for 4 h, then co-incubated with Vγ9Vδ2 T cells. Two-color and brightfield images were acquired every 2 min. Acquisition time is displayed in h: mm: ss. Scale bar represents 50 µm.
