# Supplementary figures and images for: Combining Vγ9Vδ2 T Cells with a Lipophilic Bisphosphonate Efficiently Kills Activated Hepatic Stellate Cells
Source: Front Immunol. 2017 Oct 24;8:1381. doi: 10.3389/fimmu.2017.01381 (PMC5661056; doi:10.3389/fimmu.2017.01381)

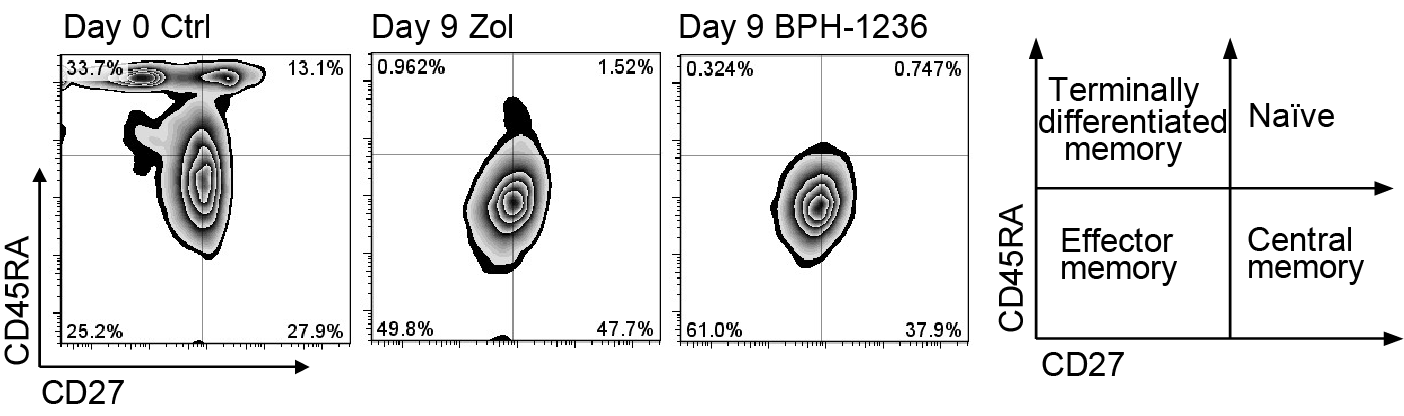

Supplement: Figure S1 — Effector memory phenotype of Vγ9Vδ2 T cells stimulated by zoledronate or BPH-1236. Vγ9Vδ2 T cells were assessed by flow cytometric staining for TCR Vδ2, CD45RA and CD27. Flow plots in all panels are representative of at least three independent experiments. [file image_1.tif]

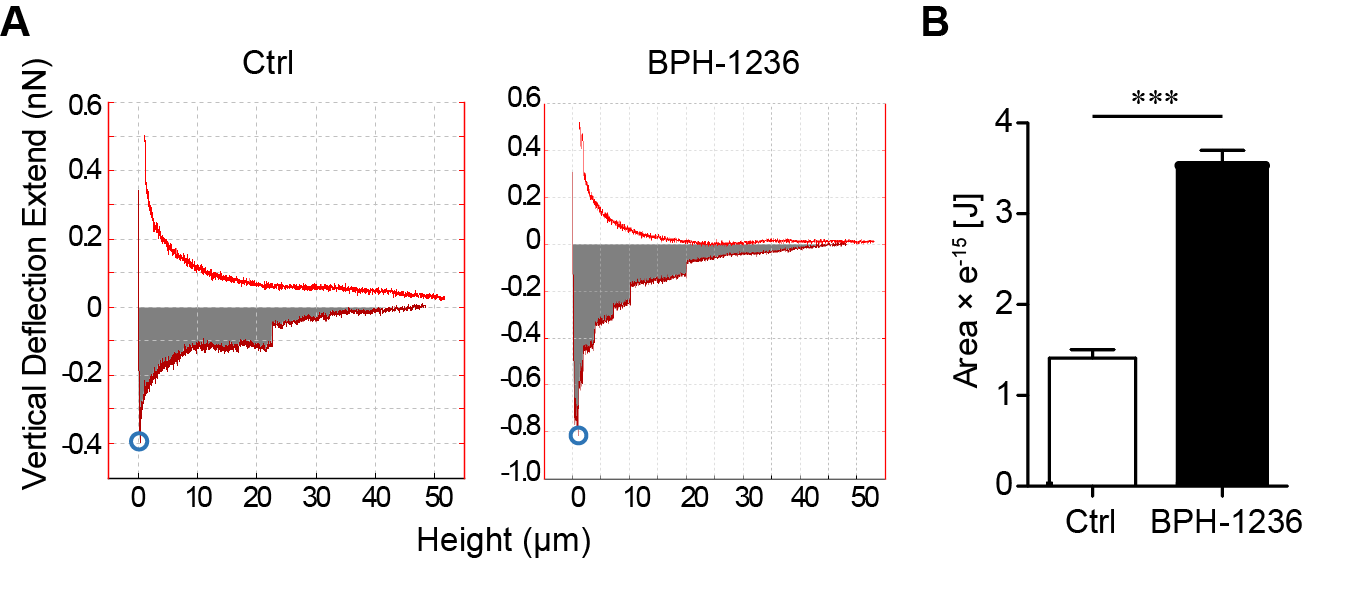

Supplement: Figure S2 — Atomic force microscopy (AFM) force curve assay. (A) Two representative force curves showing maximum adhesion forces (blue circles) between individual Vγ9Vδ2 T cell and LX-2 cell untreated (left) or treated (right) with BPH-1236 and the corresponding work (shaded area) required for full separation between the two cells. (B) Histograms of work between the two cells with or without BPH-1236 treatment. Each group contains at least 50 data points from five pairs of cells with 10 cycles. ***P < 0.001. [file image_2.tif]

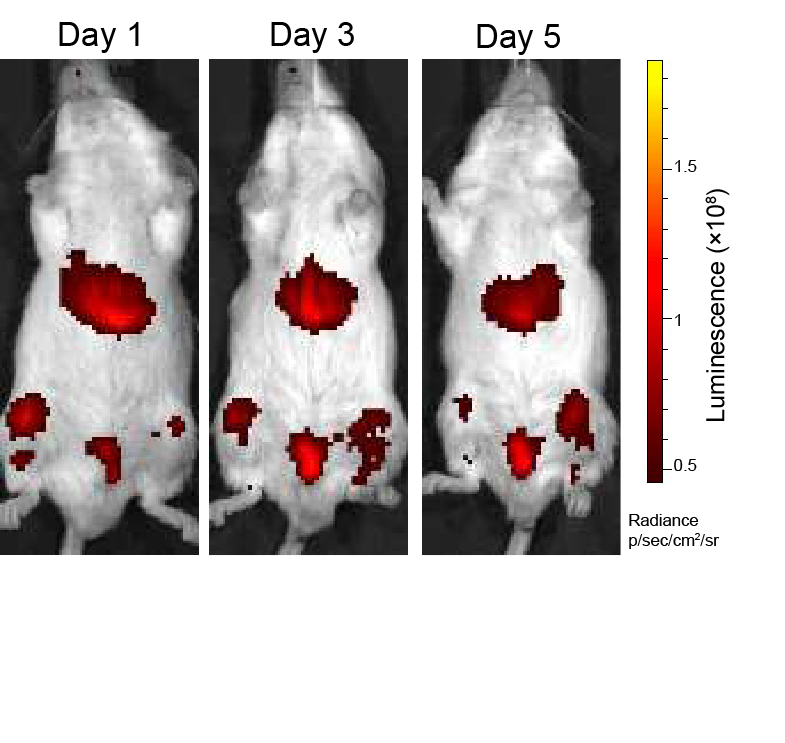

Supplement: Figure S3 — Homing behavior of Vγ9Vδ2 T cells in Rag2−/−γc−/− mice. Vγ9Vδ2 T cells were labeled with XenoLight DiR, and monitored with the IVIS imaging system on day 1, 3, 5 after i.v. adoptive transfer (n = 3). One of the two independent experiments is shown. [file image_3.tif]

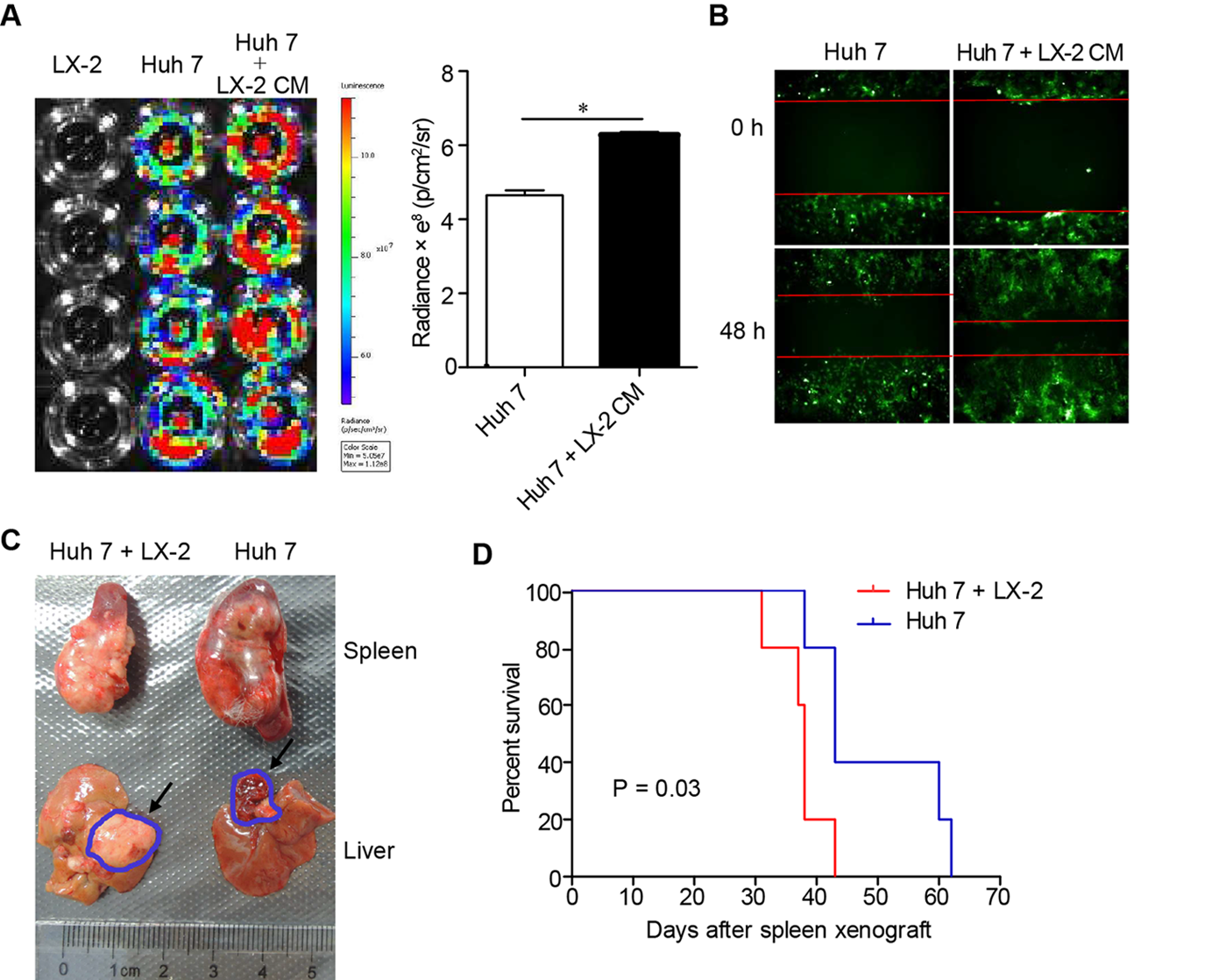

Supplement: Figure S4 — Activated HSCs promote the growth, migration, and metastasis of human liver cancer cells. (A) The growth of Huh 7/Luc cells under the control medium or LX-2 cells conditioned medium (CM) for 72 h, as determined by IVIS imaging. Data are presented as mean ± SEM of four replicates from a representative experiment of two independent experiments. *P < 0.05. (B) Representative micrograph of the areas between scratch fronts after 48 h. The scratched Huh 7/Luc cells were treated with the control medium or LX-2 cells condition medium for 48 h. Data are presented of six replicates from a representative experiment of two independent experiments. (C) Representative images of liver metastasis in Huh 7/LX-2 cells or Huh 7 cells spleen xenografts (n = 5 per group). 1 × 10^6^ Huh 7 cells and 5 × 10^5^ LX-2 cells or 1 × 10^6^ Huh 7 were injected into spleen of Rag2−/−γc−/− mice at day 0, and livers/tumors were harvested at day 43. Black arrows and blue circles highlight the tumors in liver. (D) Survival rate in Huh 7/LX-2 cells or Huh 7 cells spleen xenograft mice; n = 5 per group. [file image_4.tif]
